# Transcriptome responses to *Ralstonia solanacearum* infection in tetraploid potato

**DOI:** 10.1016/j.heliyon.2025.e41903

**Published:** 2025-01-10

**Authors:** Zhuo Chen, Shunwei Shao, Xi Zhu, Yu Zhang, Zhendong Lan, Hui Jin

**Affiliations:** a Key Laboratory of Hainan Province for Postharvest Physiology and Technology of Tropical Horticultural Products, South Subtropical Crops Research Institute, Chinese Academy of Tropical Agricultural Sciences, Zhanjiang, China; b Key Laboratory of Tropical Fruit Biology, Ministry of Agriculture and Rural Affairs of China, Zhanjiang, China; c College of Horticulture and Forestry Science, Huazhong Agricultural University, Wuhan, China

**Keywords:** Potato, *Ralstonia solanacearum*, RNA-Seq, Disease resistance, Differentially expressed genes

## Abstract

Potato (*Solanum tuberosum*) is an important global food source, the growth of which can be severely impacted by *Ralstonia solanacearum* bacterial infection. Despite extensive research, the molecular mechanisms of potato resistance to this pathogen are imperfectly known. Huashu No. 12, a tetraploid potato genotype, is highly resistant to *R. solanacearum*. We inoculate Huashu No. 12 and Longshu No. 7 (highly susceptible to *R. solanacearum*) with *R. solanacearum* to compare disease resistance in these two potato varieties. Huashu No. 12 has significantly higher resistance to *R. solanacearum* infection than Longshu No. 7, with increased lignin content, and an abundance of callose and strong autofluorescence in the phloem sieve tube. Enzymes (e.g., superoxide dismutase, catalase, peroxidase, phenylalanine ammonia-lyase, and polyphenol oxidase) contribute to *R. solanacearum* resistance in Huashu No. 12. Transcriptome sequencing reveals 659 differentially expressed genes between the two varieties, with the ethylene responsive factor family containing the most differentially expressed genes. Gene ontology and KEGG analyses provided further insights into the genetic basis and molecular mechanisms underlying plant defense against *R. solanacearum* disease. By demonstrating the importance of enzymes and differential gene expression in Huashu No. 12 resistance to *R. solanacearum* infection, the breeding of disease-resistant potato becomes increasingly feasible.

## 1 Background

Potato (*Solanum tuberosum*), the world's third most-important food crop, is vulnerable to infection by *Ralstonia solanacearum*, one of the most aggressive of bacterial pathogens to infect it. This bacterium has a wide host range (it can infect more than 450 plant species in 54 families), and causes enormous losses in food production [1]. In China, *R. solanacearum* disease has been reported from 30 provinces, but it is more prevalent in more southern and eastern areas than northern and western areas [2].

The complex physiology and adaptability of this bacterium enable it to survive in soils for several years, which complicates its eradication [3]. After entering plants through damaged root tissues, it rapidly colonizes the xylem, and then produces excess exopolysaccharides that prevent water flow. Plants consequently wilt and eventually die. Because no effective prevention measures control this disease [4], the breeding of suitably resistant crop cultivars is considered to be an appropriate management strategy [5].

In plants, the cell wall functions as the primary barrier to prevent invasion by pathogens. During early stages of infection, the plant synthesizes lignin and callose, which limit pathogen spread throughout plant tissues. Activities of phenylalanine ammonia-lyase (PAL), peroxidase (POD), polyphenol oxidase (PPO), catalase (CAT), and superoxide dismutase (SOD) then concurrently change, promoting lignification of the cell wall and inhibiting pathogen invasion [6].

One approach to better understand the mechanisms controlling plant–pathogen interactions is to integrate omics data with other information. Transcriptome sequencing, in particular, is increasingly used to study disease-resistance mechanisms in plants [7]. In tomato plants, RNA sequencing and reverse transcription quantitative PCR analyses revealed a significant upregulation of pathogenesis-related protein (SlPR-STH2) genes, due to the overexpression of SlWRKY30 [8], the resistance of H7996 tomato to wilt is, to a certain extent, mediated by the presence of inducible phenolic compounds within the xylem sap [9]. In tobacco plants, a comparative transcriptomic analysis of *R. solanacearum*-inoculated roots in C244 and C035, as well as C010 and C035, led to the identification of an additional 33 candidate genes. Among these, Nitab4.5_0007488g0040, a member of the pathogenesis-related protein 1 (PR-1) family, was found to positively regulate resistance against bacterial wilt [10].

When the late-blight resistant potato genotype SD20 was subjected to exogenous ethylene application for gene expression profiling, specific transcriptional responses to the treatment were revealed; a total of 1226 ethylene-specific differentially expressed genes (DEGs) with significantly differentially expressed transcription factors, kinases, defense enzymes, and disease resistance-related genes were identified [11]. Molecular mechanisms underlying salt tolerance in the potato variety Longshu No. 5 were also identified using NaCl treatments and time-course RNA sequencing [12].

Wild-type potato *S. commersonii* exhibits resistance to biotic and abiotic stressors [13]. Microarray analysis revealed the response of a resistant genotype against *R. solanacearum* that during early infection stages, photosynthetic processes were greatly inhibited, and that several genes encoding proteins related to reactive oxygen species production were significantly differentially expressed [14]. Type III Secretion System Effectors (T3Es) are crucial in the interaction between potato and *R. solanacearum*, with avirulent T3Es recognized by host resistance proteins helping to uncover resistance mechanisms [15].

RNA-seq analysis was used to investigate its transcriptional response to *R. solanacearum*, revealing that the number of DEGs was significantly greater than observed in susceptible controls. Moreover, approximately half of the up-regulated genes were involved in plant–pathogen interactions [16]. More recent research on potato bacterial wilt has focused on wild-type varieties than on cultivars, largely because of low levels of resistance in tetraploid varieties. To better understand molecular interactions between potato and *R. solanacearum*, we expose the tetraploid Huashu No. 12—a variant that is highly resistant to bacterial wilt—to *R. solanacearum*, to identify defense-related pathways.

## 2 Materials and methods

### 2.1 Plant materials and bacterial strains

Two potato cultivars, Longshu No. 7 (susceptible to *R. solanacearum*) and Huashu No. 12 (resistant to *R. solanacearum*), were grown in a greenhouse in the pots at 18–25 °C under a 16 h/8 h (light/dark) photoperiod. Plants of 3–4 weeks of age were used in assays.

The strain of *R. solanacearum* P2 (phylotype Ⅰ) was isolated from infected potato plants in western Guangdong, China, and used to inoculate tested plants. Bacteria were grown overnight in a nutrient-broth medium containing glucose (10 g/L), peptone (5 g/L), and beef extract (3 g/L), at pH 7.0 and 28 °C, with centrifuged at 4.5 g. Prior to plant inoculation, bacterial concentration was adjusted to 10^7^ colony-forming units ml. Control plants were inoculated with sterile water. Each test group consisted of 10 plants, with three replicates for each bacterial-resistance test.

### 2.2 Plant inoculation and disease rating

To investigate the incidence and severity of *R. solanacearum* infection, potato plants were inoculated using the root injury methods previously described by Hayward [17], after the roots were wounded with sterilized blade, 20 mL of *R. solanacearum* suspension was added into each flask and sterile water was used as blank control. Inoculations were performed with the same bacterial concentration and at a consistent time each day, with incidence monitored daily for 21 days. Plants were assessed for disease symptoms using a disease score ranging from 0 to 9 (0, no wilted leaves; 1, 1 or 2 wilted leaves; 3,33 % of leaves wilted; 5, 50 % of leaves wilted; 7, 66 % of leaves wilted; 9, 100 % of leaves wilted or dead) [18].

The disease index (DI) was calculated, where DI = Σ (number of the plants with a specific DI × DI)/(total number of inoculated plants × 9) × 100 %. The relative value (RV) of the disease was calculated, where RV = (DI of susceptible control − DI of the tested plant material/(DI of susceptible control) × 100 %. Resistance was divided into five grades according to RV: 1, highly resistant (RV > 75 %); 3, medium resistance (75 % ≥ RV > 55 %); 5, resistant (55 % ≥ RV > 35 %); 7, susceptible (35 % ≥ RV > 15 %); and 9, highly susceptible (RV < 15 %) [19].

### 2.3 Differential staining of stem base cross section

#### 2.3.1 Lignin detection

To visualize lignin deposition in plant stems—a key component of the plant's defense response [20]—a 1 cm section of the base of the plant stem was removed and sliced at two time points during the experiment (0 and 7 days post-infection (dpi)). Slices were first immersed in a solution of 5 % phloroglucinol in 95 % ethanol for 2–5 min, then treated with concentrated hydrochloric acid for 3–6 min, and finally rinsed with water and observed under a microscope. Images were captured for later analysis.

#### 2.3.2 Callose detection

At 2 dpi, sections of the plant stem were treated with aniline blue stain, a fluorescent dye that binds to chitin, a component of fungal cell walls. After staining, sections were immediately observed under an ultraviolet fluorescence microscope [21].

### 2.4 Enzyme activity determination

At four time points during the experiment (0, 24, 48, and 72 h post-inoculation (hpi)), a 1 cm section of the stem located 1 cm from the root was harvested, washed with distilled water, and immediately placed into liquid nitrogen, then stored at −80 °C. To ensure statistical rigor, five plants were selected at each time point, and three biological replicates were performed. Proteins were extracted from frozen stem tissues by grinding them into a fine powder using liquid nitrogen, and then mixing the powder with 9 × the volume of phosphate buffer (0.1 mol/L, pH 7–7.4). The mixture was centrifuged at 4 °C for 15 min at 16,100 g and the resulting supernatant was collected.

To assess the activities of enzymes involved in plant defense, reactions were performed according to manufacturer instructions using a commercial assay kit (Nanjing Jiancheng Bioengineering Co., Ltd.). The reaction solution was measured using an ultraviolet spectrophotometer and Tecan Spark microplate reader, with absorbance values substituted into a formula to calculate SOD, CAT, POD, PAL, and PPO activities. CAT enzymatic activity is expressed as the amount of enzyme required to decompose 1 μmol hydrogen peroxide per minute at pH 7.0, and is presented in U/mg protein. PAL, PPO, and POD enzymatic activities are expressed as increases of 0.01 absorbance units at 290, 398, and 470 nm per minute, respectively; all are presented in U/g. SOD activity is expressed as the amount of enzyme required to inhibit 50 % of nitroblue tetrazolium hotochemical reduction, and is presented in U/g.

### 2.5 Transcriptome analysis

RNA extraction was performed using commercial kits (Beijing Huayueyang Biotechnology Co., Ltd), with RNA sample purity, concentration, and integrity assessed using multiple methods, including Nanodrop, Qubit 2.0, and Agilent 2100. Reverse transcription of cDNA was performed using a commercially available kit (Novizan Biological Co., Ltd). Data analysis was performed using the bioinformatics pipeline provided by BMKCloud (www.biocloud.net).

Raw data obtained from the sequencing platform were subjected to offline filtering to obtain clean data, which were then compared with a specified reference genome sequence to generate mapped data to perform library quality assessments such as insert fragment length tests and random inspections. Structural level analysis, including alternative splicing analysis, new gene mining, and gene structure optimization, was performed on mapped data.

Differential expression analysis was conducted using gene expression levels in sample groups, followed by DEG functional annotation and enrichment analysis.

### 2.6 qRT-PCR validation

To confirm the accuracy and reliability of sequencing results, 10 DEGs were selected and subjected to quantitative real-time polymerase chain reaction (qRT-PCR) validation. The relative expression levels of each target gene were calculated using the 2^−ΔΔCt^ method. To perform qRT-PCR, primers for both the reference gene 18S rRNA (actin gene) and target genes were synthesized by Sangon Biotech Co., Ltd. qRT-PCR amplification was then performed using the Real Universal Color Fluorescence Quantitative Premix Kit (Tiangen Biotech Co., Ltd.) following manufacturer instructions.

All primers were designed using Primer Premier 5.0. By confirming the differential expression of selected genes through qRT-PCR analysis, the accuracy and reliability of RNA sequencing data can be validated to ensure that subsequent analyses are based on a solid foundation of high-quality data.

### 2.7 Statistical analysis

Statistical analysis was performed using Microsoft Excel 2019 and IBM SPSS Statistics 26 software. Graphs were generated using Origin 2022 and Adobe Illustrator 2021.

## 3 Results

### 3.1 Identification of *R. solanacearum* resistance inoculation

**Fig. 1:**
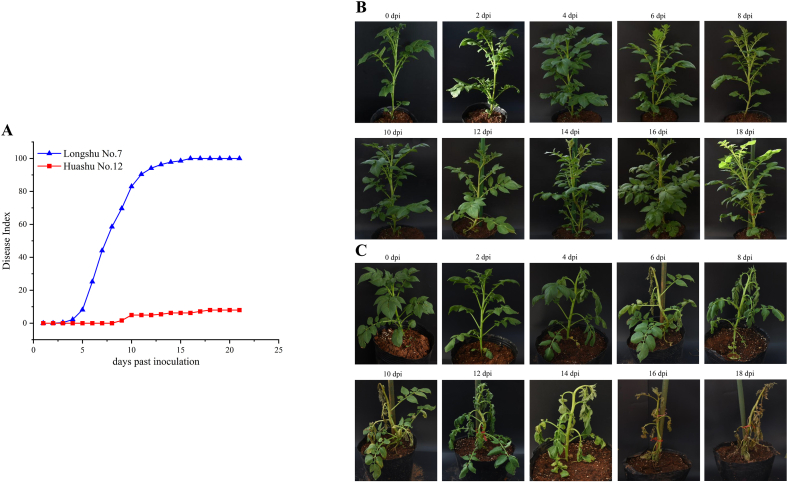
A, disease index investigation following inoculation with *Ralstonia solanacearum* to Huashu No. 12 and Longshu No. 7; and incidence of *R. solanacearum* at 0, 2, 4, 6, 8, 10, 12, 14, 16 and 18 days post inoculation in B, Huashu No. 12, and C, Longshu No. 7.

To determine the resistance level of Huashu No. 12 and Longshu No. 7 to bacterial wilt, the bacterial strain P2 was inoculated into the root systems of Huashu No. 12 and Longshu No. 7 cultivars using the root-injury method. Disease progression was monitored over 21 d; DI was calculated to indicate the severity of bacterial infection. At 16 dpi, the DI of Longshu No. 7 peaked at 100 %, while that of Huashu No. 12 was 6.22 % (Fig. 1B). By 18 dpi, the DI of Huashu No. 12 had increased to 7.97 % (Fig. 1A). The RV of the DI was 92.03 %, indicating that Huashu No. 12 is a potato cultivar with high resistance to *R. solanacearum*.

At 4, 6, and 8 dpi, Longshu No. 7 plants were at grades 1 (Fig.s. 1C), 3 and 7 stages of leaf wilting, respectively. No leaf wilting was apparent in Huashu No. 12 plants throughout this time. At 12 dpi, all Longshu No. 7 leaves had wilted (grade 9), and the first few leaves had begun to wilt on Huashu No. 12 plants (grade 1). At 18 dpi, wilting occurred on ∼33 % of Huashu No. 12 leaves (grade 3; Fig. 1A). These results suggest that Huashu No. 12 is more resistant to *R. solanacearum* infection than Longshu No. 7.

### 3.2 Histological effects of *R. solanacearum* infection in potato plants

**Fig. 2:**
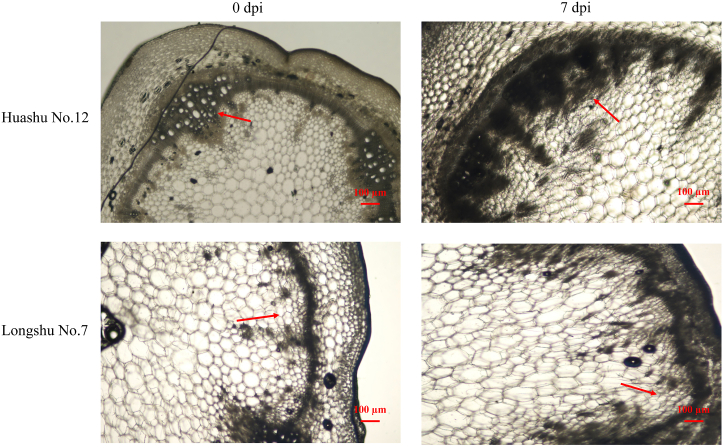
Observation of lignin contents at the stem base at 0 and 7 days post inoculation with *Ralstonia solanacearum*.

To determine the difference in lignin content between two potato varieties before and after inoculation with *R. solanacearum*, both Huashu No. 12 and Longshu No. 7 have a certain quantity of lignin when not inoculated with *R. solanacearum*, but this is greater in Huashu No. 12 (Fig. 2). At 7 dpi, the lignin content of both varieties increased, but this increase was greater for Huashu No. 12, suggesting that it may have a greater ability to produce lignin in response to *R. solanacearum* infection—a supposition consistent with its increased resistance to this pathogen.

**Fig. 3:**
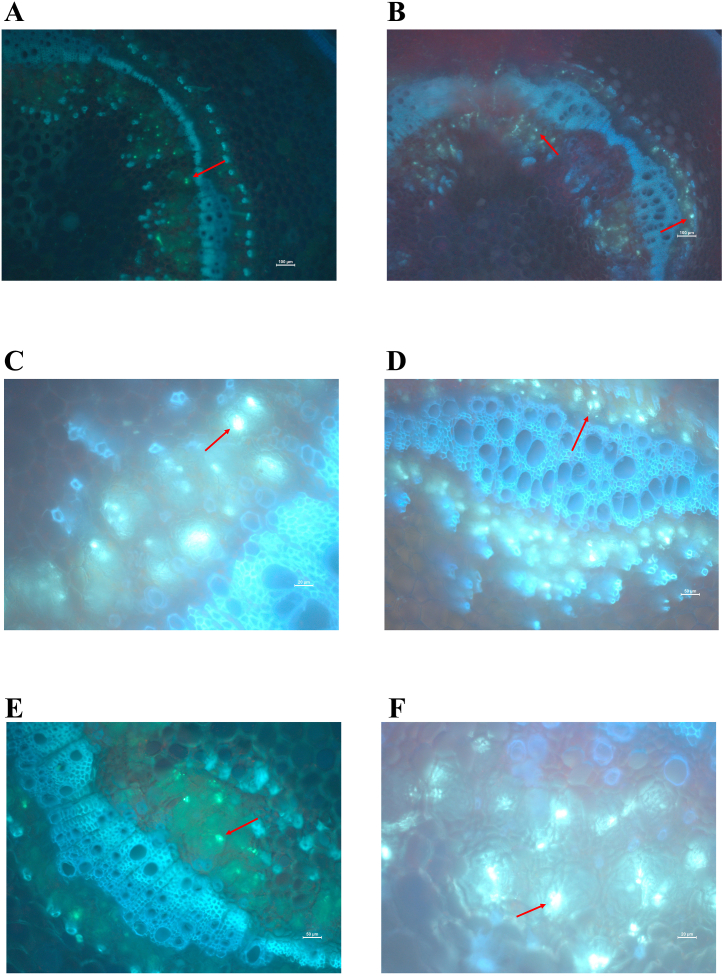
Callose distribution at the base of the stem at 2 days post inoculation with *Ralstonia solanacearum*: A, C, E, Huashu No. 12; and B, D, F, Longshu No. 7.

To assess the difference in callose position between two varieties before and after inoculation with *R. solanacearum*. Callose stained with aniline blue solution at 2 dpi. In Huashu No. 12 there was an abundance of callose in phloem sieve tubes (Fig. 3A–C, E), but in Longshu No. 7, callose filling the sieve tubes occurred in the cell-communication area between phloem tissue and cortical parenchyma cells (Fig. 3B–D, F). After inoculation with *R. solanacearum*, the defense response of Huashu No. 12 increased, leading to an increase in the number of catheter tissues with strong autofluorescence in lignified tissues (Fig. 3E). The unique location and abundance of callose in Huashu No. 12 may contribute to its increased resistance to *R. solanacearum* infection, potentially by enhancing its ability to transport defense-related molecules through phloem tissues. In the infected plants (Longshu No.7), the enhanced resulting from induced hyperplastic the enhanced quantity of conductor tissue resulting from induced hyperplastic activity was observed as a strong autofluorescence of the lignified tissue.

### 3.3 Comparative analysis of enzyme activity

**Fig. 4:**
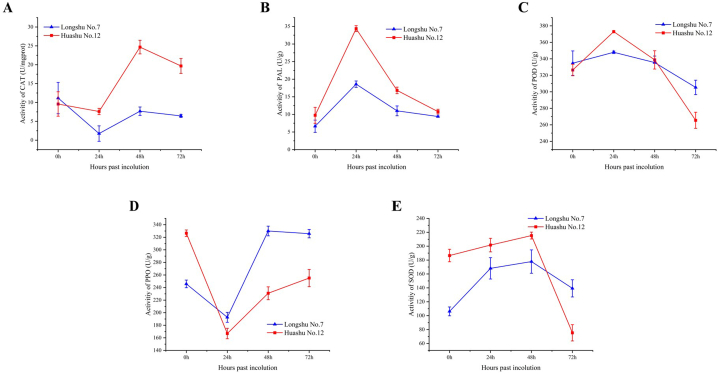
Changes in A, catalase (CAL); B, peroxidase (PAL); C, POD (phenylalanine ammonia-lyase); D, polyphenol oxidase (PPO); and E, superoxide dismutase (SOD) following inoculation with *Ralstonia solanacearum*.

Detect the changes in enzyme activities of two potato varieties within 0–72 hpi with *R. solanacearum*. CAT activities in response to *R. solanacearum* infection decreased at 24 hpi in both Huashu No. 12 and Longshu No. 7, but this decrease was greater in Longshu No. 7. The increase in CAT activity between 24 and 48 hpi was significantly higher in Huashu No. 12. CAT activity decreased between 48 and 72 hpi, but the decrease rate was slower. Overall, CAT activity was higher in Huashu No. 12, excepting at 0 hpi (Fig. 4A).

Activities of PAL and POD increased within 24 hpi, then decreased in both varieties; activities of both were greater in Huashu No. 12 (Fig. 4B and C). Over the first 24 hpi, PPO enzyme activity trended downward, more so in Huashu No. 12, before trending upward, from 24 to 48 hpi significantly faster in Longshu No. 7 than in Huashu No. 12, but thereafter the increase rate of Huashu No. 12 was slightly greater (Fig. 4D).

SOD enzyme activity increased within 48 hpi and then decreased, and was always higher in Huashu No. 12, suggesting that this variant may have a greater ability to regulate enzyme activities in response to *R. solanacearum* infection—consistent with its greater resistance to this pathogen (Fig. 4E).

### 3.4 RNA-sequencing and mapping

To preliminarily explore the molecular mechanism of Huashu No. 12's resistance to *R. solanacearum*. Using sequencing-by-synthesis technology, we sequenced 18 samples using the Illumina high-throughput sequencing platform. FASTQ format was used to assess the sequencing quality, and to obtain high-quality clean data by removing artefacts and low-quality reads. In total, we generated 125.24 GB of clean data across all 18 samples, with each sample yielding clean data of 5.87 GB in size with a Q30 base percentage of ≥90.62 % (Supplementary Table 1). Clean reads of each sample were then aligned with the potato reference genome v4.03; an alignment efficiency of 83.61%–86.86 % was achieved. The percentage of reads in clean reads ranged from 80.94% to 84.02 %.

### 3.5 Differential expression analysis

**Fig. 5:**
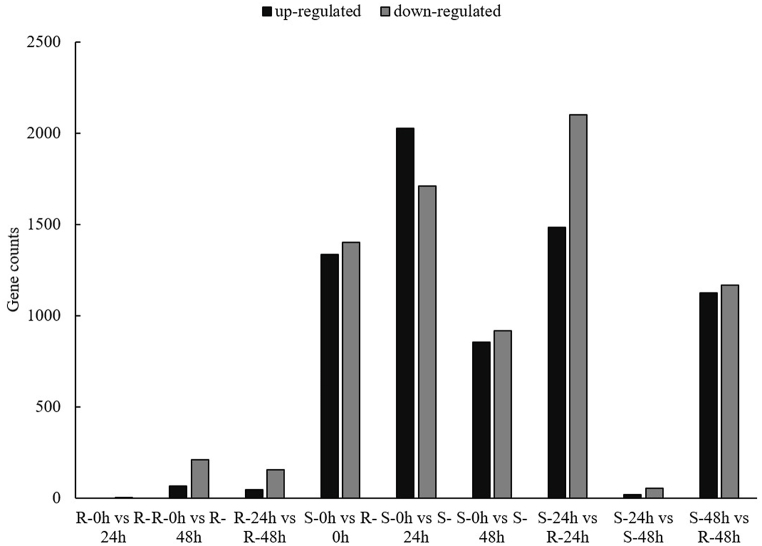
Numbers of differentially expressed genes.

**Fig. 6:**
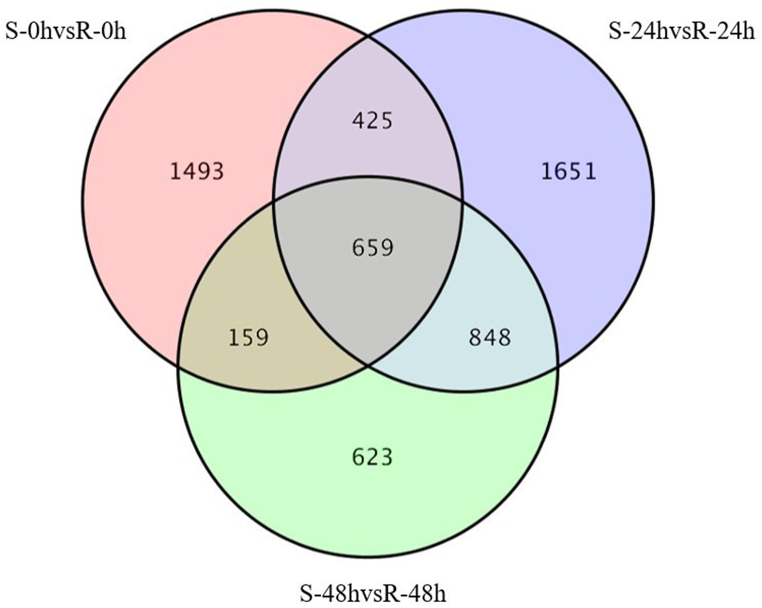
Venn diagram of differentially expressed genes (0, 24, and 48 hpi).

To gain a more comprehensive understanding of RNA-sequencing data. We used DESeq2 (1.6.3) to perform differential expression analysis between sample groups to obtain a set of DEGs between the two biological conditions. For DEG detection, a fold change (FC) ≥ 2 and false discovery rate (FDR) < 0.01 were used as screening criteria, obtained by correcting the p-value. The number of DEGs in each set is shown in Fig. 6. For Huashu No. 12, four DEGs were down-regulated compared with 24 hpi; of 199 DEGs identified at 24 hpi and 48 hpi, 44 were up-regulated and 155 were down-regulated; and of 272 DEGs identified between 0 hpi and 48 hpi, 63 were up-regulated and 209 were down-regulated (Fig. 5). For Longshu No. 7 at 24 hpi, 3736 DEGs were detected, of which 2026 were up-regulated and 1710 were down-regulated; of 72 DEGs identified from 24 hpi and 48 hpi, 18 were up-regulated and 54 were down-regulated; of 1769 DEGs identified at 48 hpi, 854 were up-regulated and 915 were down-regulated (Fig. 5, Supplementary Table 2). Of the 5858 DEGs detected in both Huashu No. 12 and Longshu No. 7, 659 were common to both varieties at 0, 24, and 48 hpi (Fig. 6). The DEGs of Huashu No. 12 is lower than that of Longshu No. 7, but the activities of enzymes such as CAT, PAL, and POD are higher than those of Longshu No. 7, indicating that these enzymes play a key role in resistance to bacterial wilt.

### 3.6 GO enrichment of DEGs

To identify significantly enriched Gene Ontology (GO) terms for the DEGs, we used R/topGO (2.18.0) and set the parameter “First Sig Nodes = 10.” Our analysis covered biological process, cellular component, and molecular function branches of GO.

**Fig. 7:**
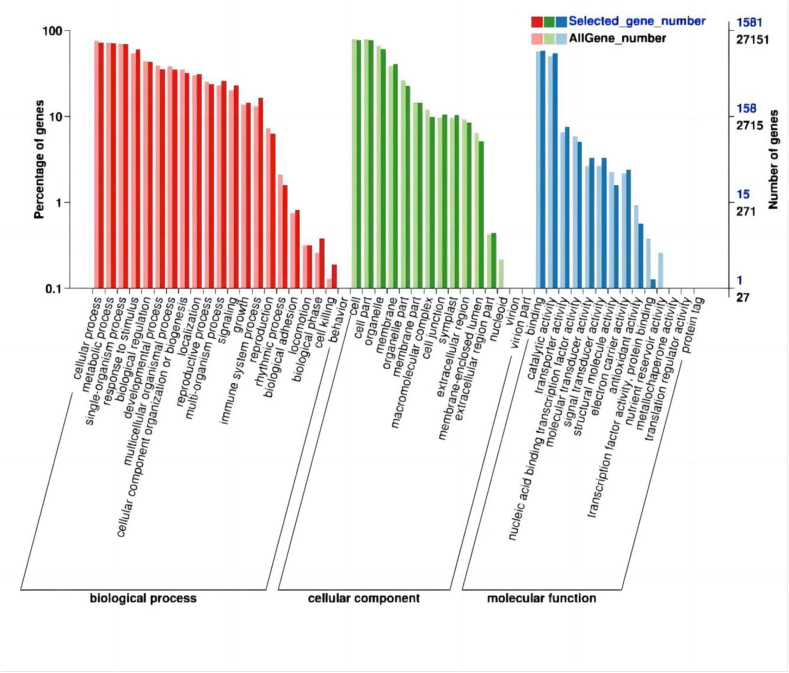
Gene ontology analysis of differentially expressed genes.

Most enriched biological process GO terms were linked to cellular process (GO:0009987, 1146 DEGs), metabolic process (GO:0008152, 1116 DEGs), and single-organism process (GO:0044,699, 1096 DEGs). For cellular components, many DEGs were annotated to cell (GO: 0005623, 1217 DEGs), cell part (GO: 0044,464,1217 DEGs), and organelle (GO:0043,226, 958 DEGs). Most enriched molecular function GO terms were related to binding (GO:0005488, 925 DEGs), catalytic activity (GO: 0003824, 856 DEGs), and transporter activity (GO: 0005215, 119 DEGs) (Fig. 7, Supplementary Table 3).

### 3.7 Kyoto Encyclopedia of Genes and Genomes (KEGG) enrichment of DEGs

**Fig. 8:**
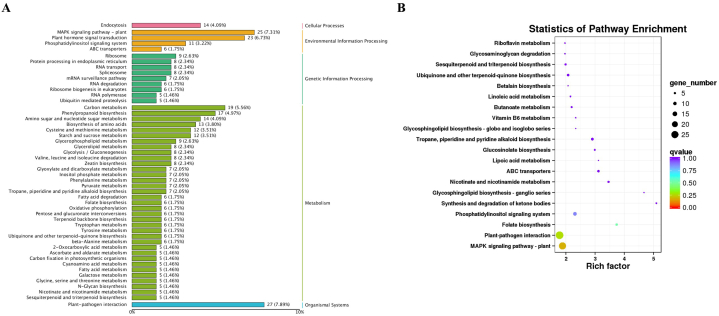
A, KEGG classification map of differentially expressed genes; B, pathway analysis of differentially expressed genes.

Kyoto Encyclopedia of Genes and Genomes orthologs (KEGG) was used to analyze DEGs in each treatment. The 6392 DEGs with KEGG annotation were assigned to 342 KEGG pathways (Fig. 8, Supplementary Table 4). Many DEGs were annotated to plant–pathogen interaction, MAPK signaling pathway-plant, plant hormone signal system, carbon metabolism, and phenylpropanoid biosynthesis (Fig. 8A). Synthesis and degradation of ketone bodies, glycosphingolipid biosynthesis-ganglio series, nicotinate and nicotinamide metabolism, ABC transporters, and lipoic acid metabolism were most significantly enriched in DEGs (Fig. 8B).

### 3.8 Differential expression of transcription factors in response to *R. solanacearum*

**Fig. 9:**
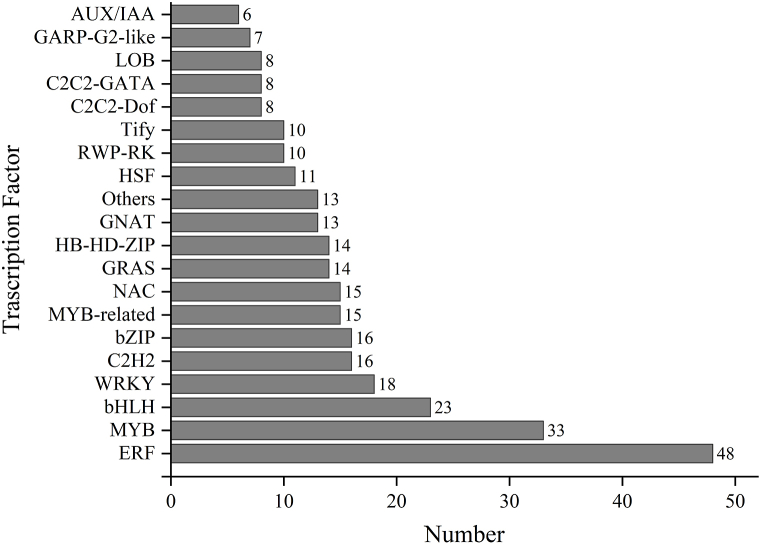
Number of transcription factors in different families that were differentially expressed following inoculation with *Ralstonia solanacearum.*

Among the 5858 genes differentially regulated by *R. solanacearum*, 419 encoded transcription factors (TFs) belonging to 63 families (Supplementary Table 5). Most TFs were ethylene responsive factor (ERF), followed by MYB, bHLH, WRKY, C2H2, bZIP, MYB-related, NAC, GRAS, HB-HD-ZIP, GNAT, and others (Fig. 9).

### 3.9 qRT-PCR verification of DEGs

**Fig. 10:**
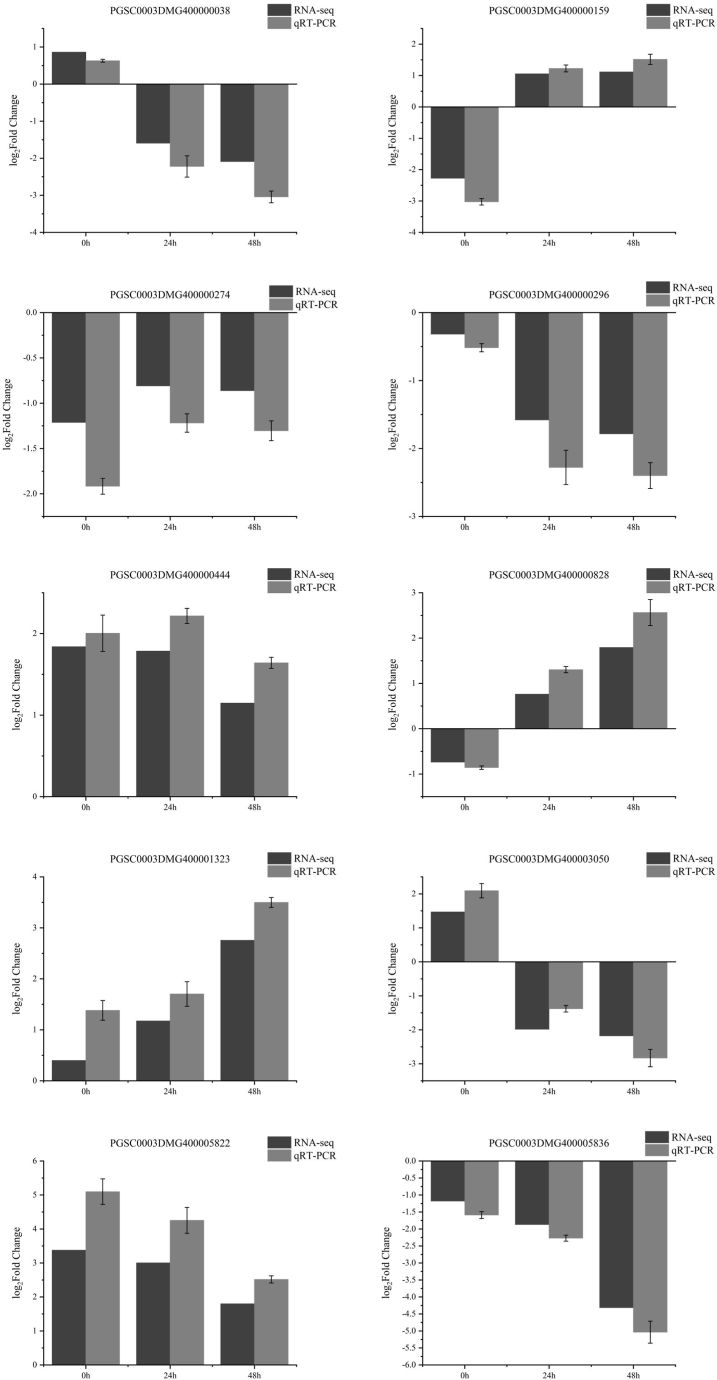
qRT-PCR verification.

To verify the validity of transcriptome sequencing results, 10 DEGs were randomly selected for qRT-PCR validation (Supplementary Table 6). The log_2_Fold Change value verified by qRT-PCR was basically consistent with transcriptome sequencing results (Fig. 10).

## 4 Discussion

### 4.1 Tetraploid potato genotype Huashu No. 12 resistance to *R. solanacearum*

In field evaluations aimed at screening potato materials for *R. solanacearum* resistance, somatic hybrids created by crossing *S. commersonii* and *S. tuberosum* had phenotypes similar to those of cultivated potato, and the F_2_ progeny had a promising transfer of *S. commersonii* beneficial traits into the cultivated background [22]. Simple sequence repeat alleles were identified for bacterial wilt resistance breeding in *S. chacoense*-somatic hybrids and backcross progenies up to the BC3 generation [23]. However, despite efforts to use somatic hybrid material, no registered cultivars have been produced [24]. We report Huashu No. 12 to be highly resistant to phylotype Ⅰ *R. solanacearum* in a 21 day DI investigation (Fig. 1), and conclude that it is an excellent candidate for development of tetraploid potato varieties with enhanced resistance to *R. solanacearum*.

### 4.2 Histological effects of *R. solanacearum* infection in potato plants

Secondary metabolites, including phenylpropanoids and biosynthesis related to cell wall synthesis, participate in the defense responses of potato against *Phytophthora infestans* by synthesizing lignin and strengthening the cell wall [25,26]. Of two potato varieties, orion and majestic, the lignin content of the more-resistant orion significantly increased after infection compared with the more-susceptible majestic variety. Increased PAL activity and lignin content improved potato tuber resistance to *P. infestans* and *Fusarium sulphureum* [27,28]. We report the lignin content of Huashu No. 12 to have increased more than that of Longshu No. 7, indicating that lignin is involved in potato resistance to *R. solanacearum*.

Inoculation of plants with endophytic bacteria confers protection against pathogenic bacteria that may be mediated through mechanisms such as cell wall reinforcement and increased lignification and callose deposition, which impede successful pathogenic bacterial colonization [29,30]. We report the callose in Huashu No. 12 to be located within and to fill the sieve tube of phloem tissue, and to be situated in the region of cellular communication between the phloem tissue and cortical parenchyma cells in Longshu No. 7. These results suggest that the different positions of callose in these cultivars may be attributed to variations in the expression of callose synthase gene following infection with *R. solanacearum*. This disparity could play an important role in resistance to *R. solanacearum* infection in Huashu No. 12.

### 4.3 Effect of *R. solanacearum* on potato defense enzyme activity

Plants have evolved sophisticated mechanisms to defend themselves against pathogenic microorganisms. Increasing the activity of resistance-related enzymes is one such strategy. In plant disease-resistance research, CAT is the most-studied enzyme [31]. Susceptible plant varieties tend to have higher levels of CAT activity than resistant varieties in the absence of *R. solanacearum* inoculation [32], consistent with our results. While CAT activity in Huashu No. 12 was initially lower than that of Longshu No. 7 at 0 hpi, it surpassed CAT activity of Longshu No. 7 at all other time points.

PAL, an important enzyme involved in the phenylpropanoid metabolic pathway, plays a crucial role in biosynthesis of secondary metabolites such as lignin and phenols. These metabolites are involved in various physiological and defense-related processes [33]. As such, PAL is considered to be an important defensive enzyme that confers protection against biotic and abiotic stresses in plants, PAL is also the first key enzyme in the lignin biosynthesis pathway [34]. We report PAL activity in Huashu No. 12 to be consistently higher than that of Longshu No. 7 following inoculation with *R. solanacearum*, especially at 24 hpi. This suggests that 24 hpi may be a critical period for potato resistance to bacterial wilt, and that PAL plays a central role in conferring resistance to *R. solanacearum* infection in potato.

PPO and POD are two commonly studied defense-related enzymes in plants that play important roles in plant immune responses to pathogenic microorganisms. These enzymes act as biocontrol agents, increasing the plant's immune response by catalyzing the production of reactive oxygen species and other signaling molecules that help to activate and regulate plant defense mechanisms [[35], [36], [37]]. Therefore, PPO and POD are regarded as essential enzymes to enhance plant resistance to disease and other stresses. We report the activity of POD to initially increase and then decrease following inoculation with *R. solanacearum*, with the amplitude of increase being greater in Huashu No. 12. Conversely, the activity of PPO first decreased and then increased, with the decrease rate being greater in Huashu No. 12. These findings suggest that regulation of POD and PPO activities varies between potato cultivars in response to *R. solanacearum* infection, indicating the importance of these defense-related enzymes in conferring resistance against bacterial wilt in potato plants.

SOD plays an important role in the ability of plant cells to resist viral infections. Accordingly, it is widely regarded to be an essential enzyme in the plant's defense machinery [38]. We report SOD activity of Huashu No. 12 to be higher than that of Longshu No. 7, and for it to first increase and then decrease.

### 4.4 Transcriptomic investigation of the impact of *R. solanacearum* on resistance to bacterial wilt in potato

Transcriptome sequencing has been increasingly used to study mechanisms underlying plant disease resistance [[39], [40], [41], [42], [43]]. A systematic investigation of the transcriptomic dynamics of Qingshu No. 9 infected with *P. infestans* was performed to identify resistance-related genes; many DEGs, including transcription factor genes, were significantly enriched in biosynthesis, plant-pathogen interaction, and photosynthesis pathways, and several genes related to disease resistance were also differentially expressed during infection [44].

We will discuss how the resistant cultivar Huashu No. 12 might have a more efficient or targeted defense response, which could explain the higher activity of these specific enzymes despite having fewer DEGs overall. These enzymes play crucial roles in oxidative stress responses and pathogen defense mechanisms:

CAT helps in detoxifying hydrogen peroxide, which can accumulate during stress responses. PAL is involved in the biosynthesis of phenylpropanoids, including those that form physical barriers and antimicrobial compounds. POD contributes to cell wall strengthening and the production of reactive oxygen species that can combat pathogens. SOD mitigates the damage caused by superoxide radicals.

We hypothesize that the resistant cultivar may have optimized its defense mechanisms to upregulate these key enzymes, resulting in effective resistance with potentially less need for widespread transcriptional changes. This efficiency could be a result of selective pressure and evolutionary adaptation, making the resistant cultivar more adept at managing *R. solanacearum* infection.

*R*. *solanacearum* infection in potato plants can trigger changes at the transcriptional level, leading to alterations in gene expression and subsequent modulation of various physiological processes that underlie the plant's defense response. We used DESeq2 (1.6.3) software to identify a set of DEGs between two biological conditions, using FC ≥ 2 and FDR <0.01 as screening criteria. A total of 5858 DEGs were detected in both conditions. GO annotation analysis revealed these DEGs to be mainly involved in cellular processes, metabolic processes, single-organism processes, cell, and organelle. KEGG pathway enrichment analysis indicated that these DEGs were mainly associated with plant–pathogen interaction, MAPK signaling pathway-plant, plant hormone signal system, carbon metabolism, and phenylpropanoid biosynthesis. We also identified 420 TFs belonging to 50 families that regulate these DEGs, with ERF, MYB, and bHLH being the most common.

## CRediT authorship contribution statement

**Zhuo Chen:** Writing – review & editing, Writing – original draft, Funding acquisition, Formal analysis, Data curation, Conceptualization. **Shunwei Shao:** Writing – original draft, Software, Methodology, Investigation. **Xi Zhu:** Resources, Project administration, Methodology, Investigation, Conceptualization. **Yu Zhang:** Writing – original draft, Visualization, Validation, Supervision, Software. **Zhendong Lan:** Writing – original draft, Visualization, Resources. **Hui Jin:** Writing – original draft, Supervision, Resources, Project administration, Data curation, Conceptualization.

## Informed consent

Informed consent was obtained from all participants involved in the study. Participants were fully informed about the purpose, procedures, potential risks, and benefits of the study, and they provided written consent prior to participation.

## Data availability statement

The original contributions presented in the study are included in the article/supplementary material. Further inquiries can be directed to the corresponding authors.

## Funding

This work was funded by the Hainan Provincial Natural Science Foundation of China (No. 323QN296, 324QN328); the Central Public-interest Scientific Institution Basal Research Fund for Chinese Academy of Tropical Agricultural Sciences (No. 1630062024011) and the Project of National Key Laboratory Tropical Crop Breeding (No. NKLTCBZRJJ3), and conflict of interest exits in the submission of this manuscript, and all authors approve the manuscript for publication.

## Declaration of competing interest

The authors declare that they have no known competing financial interests or personal relationships that could have appeared to influence the work reported in this paper.
